# Using Boosted Machine Learning to Predict Suicidal Ideation by Socioeconomic Status among Adolescents

**DOI:** 10.3390/jpm12091357

**Published:** 2022-08-24

**Authors:** Hwanjin Park, Kounseok Lee

**Affiliations:** 1 Department of Occupational & Environmental Medicine, Kangbuk Samsung Hospital, Sungkyunkwan University School of Medicine, Seoul 04763, Korea; 2 Department of Psychiatry, Hanyang University Medical Center, Seoul 04763, Korea

**Keywords:** adolescent, suicide, machine learning, risk factors

## Abstract

(1) Background: This study aimed to use machine learning techniques to identify risk factors for suicidal ideation among adolescents and understand the association between these risk factors and socioeconomic status (SES); (2) Methods: Data from 54,948 participants were analyzed. Risk factors were identified by dividing groups by suicidal ideation and 3 SES levels. The influence of risk factors was confirmed using the synthetic minority over-sampling technique and XGBoost; (3) Results: Adolescents with suicidal thoughts experienced more sadness, higher stress levels, less happiness, and higher anxiety than those without. In the high SES group, academic achievement was a major risk factor for suicidal ideation; in the low SES group, only emotional factors such as stress and anxiety significantly contributed to suicidal ideation; (4) Conclusions: SES plays an important role in the mental health of adolescents. Improvements in SES in adolescence may resolve their negative emotions and reduce the risk of suicide.

## 1. Introduction

Suicide is the leading cause of death among Korean teenagers [1]. The risk factors for suicide among adolescents can be divided into socio-demographic, mental health, and individual and family factors [2]. Psychiatric problems such as various types of violence and abuse experienced by teenagers, a family history of suicidal behavior, interpersonal difficulties, parental separation and divorce, loss of parents or straight friends, drug abuse, depression, and anxiety disorders are risk factors for suicide among adolescents [3,4].

With the improvement of computing technology, various analysis methods have been tried to increase the predictive power of diseases. Published studies on risk factors for suicide have mainly used regression analyses [5,6]. However, machine learning methods help achieve higher predictive accuracy and positive predictive value of suicide by analyzing risk factors for suicide [7]. The boosting is an algorithm that improves prediction or classification performance by combining multiple sequential weak learners as one of the machine learning ensemble techniques [8]. The gradient boosting algorithm is a predictive model belonging to the boosting family of ensemble methodologies that can perform regression analysis or classification analysis. The extreme gradient boosting (XGBoost) model has the advantage of improving prediction performance by normalizing variables to prevent overfitting [9]. It is known to have excellent predictive performance, and it can evaluate the complex associations between variables better than the existing linear model-based approaches [10,11].

Socioeconomic status (SES) describes the effect of social and economic aspects on individuals’ lives [12]. Thus, SES is defined as an individual’s position in a society, determined by an individual’s power, prestige, and ability to control resources.

SES is a significant factor affecting individuals’ life satisfaction, mental health, emotional development, and physical development. It also significantly affects one’s psychological health apart from their demographic background [13,14].

In this study, the risk factors for suicide were identified using the data from the Korea Youth Risk Behavior Web-based Survey (KYRBWS) conducted by the state for Korean adolescents. The associations between risk factors were also examined according to SES. First, risk factors were checked according to suicidal ideation (SI). Then using these variables, a decision tree algorithm named extreme gradient boosting (XGBoost) was used to check the accuracy of adolescent suicidal ideation prediction according to SES level. Finally, the influence of factors contributing to SI was explored.

## 2. Materials and Methods

### 2.1. Study Population

The KYRBWS is an anonymous self-report survey administered to middle- and high-school students to better understand the health behaviors of Korean teenagers. The Ministry of Education, the Ministry of Health and Welfare, and the Korean Centers for Disease Control and Prevention have been performing a government-approved statistical survey since 2005 (approval number 117058). This study was approved by the Institutional Review Board of Kangbuk Samsung Hospital, Seoul, Korea (KBSMC 2022-07-003).

The 2020 KYRBWS data were used for this study. The survey generated a national sample of middle and high school students till April 2020. Sample schools were initially extracted for each area and school type using a stratified extraction approach with permanent random numbers. In 2020, the sample class was polled for all pupils, and 57,925 youths from 800 schools (400 middle and high schools each) in 17 cities and provinces around the country were included. Overall, 54,948 adolescents participated, yielding a 94.9% participation rate. The data were acquired using unique numbers that included no personal information, and the respondent’s confidentiality was rigorously protected. We analyzed all data obtained from 54,948 adolescents.

### 2.2. Measures

#### 2.2.1. Demographic Variables

The demographic characteristics were sex (male or female), academic performance in the past year (evaluated over 5 levels), and SES.

#### 2.2.2. Suicidal Ideation

Participants were asked, “Have you ever felt that you were willing to die?” to which they had to answer yes or no.

#### 2.2.3. Mental Health-Related Variables

Subjective physical health; usual stress level; episodes of feeling sad or hopeless of sufficient intensity to hinder performing daily activities that lasted for ≥2 weeks in the previous year; feelings of happiness; violence against friends, seniors, or adults in the previous year; and Generalized Anxiety Disorder-7 (GAD-7) scores were the mental health-related variables considered in this study. In 5 stages, participants’ subjective health state, usual stress level, and feelings of happiness were assessed. The Korean version of the GAD-7 was used to assess anxiety [15].

#### 2.2.4. Health-Related Behavior

Health-related behavioral factors such as drinking, smoking, drug usage, and sexual activity were used. Respondents were asked how many times per month they drank and/or smoked. Substance misuse was evaluated by asking if they used drugs or substances regularly, except for therapeutic purposes.

### 2.3. Data Processing and Machine Learning

Respondents were divided into 2 groups based on whether they had SI, and the features of each group were examined. For continuous variables, a *t*-test was used, and for categorical variables, a chi-square test was used. SPSS (version 27; IBM Corporation, New York, NY, USA) was used for the *t*-test, ANOVA, and chi-square tests. Statistical significance was set to <0.05 for a 2-sided test.

After the general characteristics of the participants were analyzed, machine learning analysis was performed. Gradient boosting algorithms learn until they reach the specified number of trees and reduce error by iterative learning. The XGBoost method is based on a gradient boosting algorithm. Gradient boosting minimizes errors by applying the gradient descent method to boosting algorithm using a combination of several weak learners. The XGboost method uses a decision tree as a weak learner. General gradient boosting learns by increasing the weight sequentially, XGBoost learns in parallel. XGBoost is extensively used in several fields because given its benefits of fast learning and classification and excellent overfitting regulation; it is often more efficient than conventional tree analyses [16]. In this study, XGBoost analysis was performed using XGBclassfier. For the analysis, data were divided into 75% of the training dataset and 25% of the test dataset. After training using the training dataset with a 5-fold cross-validation of Scikit Learn, the results applied to 25% of the test data were presented. The prevalence of SI among the study participants was 10.9%, which may result in biased results for multiple groups [17]. Thus, using the synthetic minority over-sampling technique (SMOTE), the SI data within the training dataset were oversampled and the non-suicidal data were under sampled. The no SI and the SI groups were matched for participant count, and training was then performed. SMOTE is the most popular technique for solving data imbalance-related bias in machine learning [18].

The performance of the predictive model was presented in several measures, such as sensitivity, specificity, positive predictive value, negative predictive value, accuracy, and area under the curve (AUC). The importance of each variable in the XGBoost analysis was presented using the F score. The XGBoost analysis was used by Google Colab (https://colab.research.google.com access on 7 July 2022).

## 3. Results

### 3.1. General Characteristics of the Suicidal Ideation

Of the 54,948 participants, 5979 (10.9%) had SI in the past year and 48,969 (91.1%) did not. In the SI group, 72.6% of the participants experienced feeling sad or hopeless for ≥2 weeks within the past year, whereas only 19.4% did so in the no-SI group (*p* < 0.001). In the SI group, 74.5% of the participants experienced severe stress in daily life (level 4 or 5), which is significantly higher than 29.0% in the no-SI group (*p* < 0.001). In the SI group, 28.8% of the participants experienced feeling very happy or somewhat happy (level 4 or 5) compared to 68.3% in the no-SI group (*p* < 0.001).

A significantly higher proportion of participants underwent treatment because of physical or psychological violence in the SI group (4.7%) than in the no-SI group (0.9%; *p* < 0.001). The mean GAD-7 score was 8.84 ± 5.60 and 3.30 ± 3.78 for the SI and the no-SI group, respectively (*p* < 0.001). In the SI group, 18.9% perceived their subjective health as bad or very bad, which was significantly higher than in the no-SI group (6.2%; *p* < 0.001).

A significantly higher proportion of participants reported drinking for >6 days a month in the SI group (*p* < 0.001). Furthermore, a greater proportion of participants reported not smoking in the no-SI group (96.1%) than in the SI group (90.9%). A significantly higher proportion of participants reported sexual experiences in the SI group (9.5%) than in the no-SI group (*p* < 0.001); a similar observation was made for substance abuse rate (2.9% vs. 0.5%, *p* < 0.001). In the SI group, 41.6% of the participants had low-to-medium or low academic performance, which was higher than in the no-SI group (32.2%; *p* < 0.001). Of all participants in the SI group, 5.2% had a low SES compared to 2.0% of those in the no-SI group (*p* < 0.001; Table 1).

### 3.2. XGBoost Models by Socioeconomic Status and Prediction of Suicidal Ideation

The XGBoost analysis was performed to predict SI. After training the prediction model with training data, the results with training data and test data were presented. The XGBoost model showed good performance with AUC values of 0.773 in the high SES group, 0.846 in the medium SES group, and 0.781 in the low SES group. Generally, an AUC value of 0.5 indicates no discriminative value, whereas AUC values of ≥0.75 are clinically useful [19].

According to the confusion matrices, 81 of 140 participants in the high SES group and 1119 of 1371 participants in the no-SI group were predicted to have SI. The performance metrics of the model in the high SES group were as follows: accuracy = 0.794, sensitivity = 0.579, = specificity = 0.816, positive predictive value = 0.243, negative predictive value = 0.950, and F1 score = 0.343.

With regard to the medium SES group, 994 of 1300 participants in the SI group and 8276 of 10,609 participants in the no-SI group were predicted to have SI. The performance metrics of the model in the medium SES group were as follows: accuracy = 0.778, sensitivity = 0.765, specificity = 0.780, positive predictive value = 0.299, negative predictive value = 0.964, and F1 score = 0.430.

In the low SES group, 54 of 89 participants in the SI group and 185 of 230 participants in the no-SI group were predicted to have SI. The performance metrics of the model in the low SES group were as follows: accuracy = 0.749, sensitivity = 0.607, specificity = 0.804, positive predictive value = 0.545, negative predictive value = 0.841, and F1 score = 0.575 (Table 2).

### 3.3. Decision Tree of Suicidal Ideation by XGBoost

**Figure 1:**
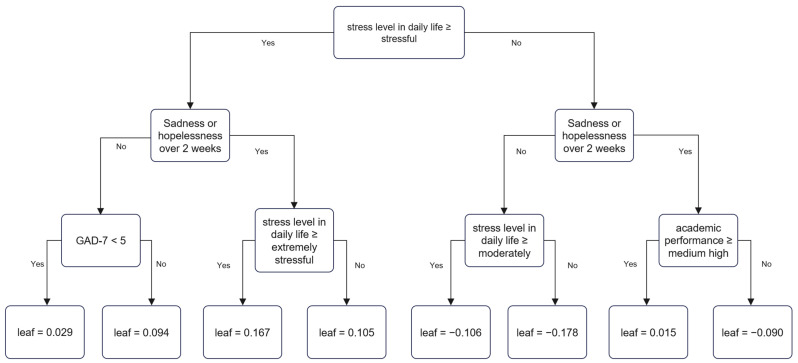
Decision tree of suicidal ideation in high socioeconomic status group by XGBoost.

In the tree structure of XGBoost, the higher the node, the more important the variable. As the tree continues to be separated, the characteristics of each node accumulate, and the probability of SI changes. In the high SES group, perceived levels of stress, sadness, or hopelessness over 2 weeks, GAD-7 score, and academic performance influenced SI as follows. Among these variables, when the stress level was more than stressful, symptoms of sadness or hopelessness were present for over 2 weeks, and when the stress level was extreme, the prediction score was 0.167, which was most strongly associated with SI. Conversely, when the stress level was moderate or less when no sadness or hopelessness was experienced over 2 weeks, and when the stress level was less than minimal, the prediction score was −0.178, which was the least strongly associated with SI (Figure 1).

**Figure 2:**
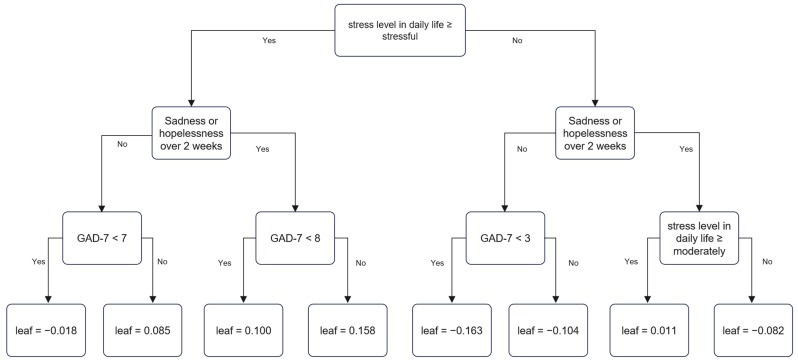
Decision tree of suicidal ideation in the medium socioeconomic status group by XGBoost.

In the medium SES group, perceived stress level, sadness or hopelessness over two weeks, and GAD-7 score were associated with SI. Stressful or extremely stressful experiences, sadness or hopelessness over two weeks, and a GAD-7 score of ≥8 yielded a prediction score of 0.158, which represented the strongest association with SI. Conversely, if the stress level was moderate or less, no symptoms of sadness or hopelessness were experienced over two weeks, and the GAD-7 score was <3, the prediction score was −0.163, which represented the weakest association with SI (Figure 2).

**Figure 3:**
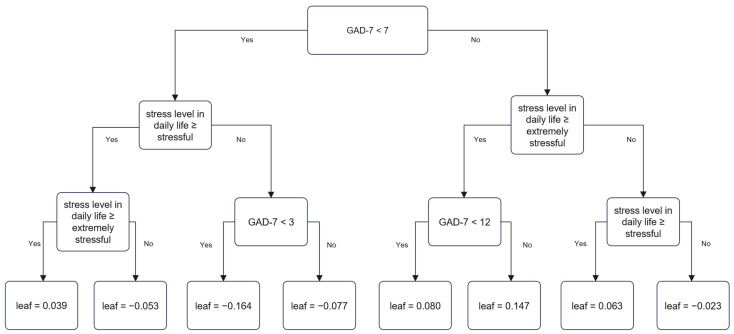
Decision tree of suicidal ideation in the low socioeconomic status group by XGBoost.

In the low SES group, GAD-7 score and perceived stress level were associated with SI. A GAD-7 score ≥7, extreme stress level, and a GAD-7 score of ≥12 yielded a prediction score of 0.147, which showed the strongest association with SI. Conversely, a GAD-7 score <7, a stress level lower than stressful, and a GAD-7 score <3 yielded a prediction score of −0.164, which represented the weakest association with SI (Figure 3).

### 3.4. Decision Tree of Suicidal Ideation by XGBoost

Of the 54,948 participants, 6039 (11.0%) were in the high SES group, 47,634 (86.7%) were in the medium SES group, and 1275 (2.3%) were in the low SES group. The proportion of female students in the medium SES group was 49.4%, which was higher than that in the total sample (48.4%, *p* < 0.001).

The proportion of participants who experienced sadness or hopelessness for ≥2 weeks within the past year was 43.7% in the low SES group, which was higher than that in the high SES group (22.4%; *p* < 0.001). Similarly, 53.5% of the participants in the low SES group experienced severe stress in daily life (at level 4 or 5) as compared to 28.0% in the high SES group (*p* < 0.001). In the low SES group, 40.6% of the participants reported feeling very or somewhat happy (at level 4 or 5), which was lower than in the high SES group (76.2%) and the medium SES group (63.1%; *p* < 0.001).

In the low SES group, 4.1% of the participants underwent treatment because of physical or psychological violence as compared to 1.3% in the entire population (*p* < 0.001). The mean GAD-7 score was 6.02 ± 5.86 in the low SES group, 3.12 ± 4.30 in the high SES group, and 3.95 ± 4.31 in the medium SES group. These data show that the GAD-7 score was significantly higher in the low SES group than in the other groups (*p* < 0.001).

In the low SES group, 16.7% perceived their subjective health as bad or very bad, which was significantly higher than in the high SES group (4.4%; *p* < 0.001). The low SES group reported the highest proportion of participants drinking >6 days a month and smoking >10 days a month than the medium and high SES groups (*p* < 0.001). The proportion of participants reporting sexual experiences in the low SES group (11.1%) was significantly higher than in the other two groups (*p* < 0.001). The low SES group also reported a higher proportion of participants engaged in substance abuse (2.4%) than did the high SES (1.0%) and medium SES groups (0.7%; *p* < 0.001). In the low SES group, the proportion of participants with low-to-medium or low academic performance was 65.7%, which was higher than that in the high SES (21.1%) and medium SES groups (33.8%; *p* < 0.001). The proportion of participants reporting SI in the low SES group was 24.3%, which was higher than that in the high SES (8.6%) and medium SES groups (10.8%; *p* < 0.001, Table 3).

## 4. Discussion

In the past, attempts have been made to predict suicide using machine learning methods. Although the method has been improved, there is a limitation that the prediction rate is not significantly improved [20]. However, in previous studies, when the same sample was analyzed, the prediction rate was increased depending on the analysis method [21,22]. In this study, using the gradient boosting algorithm, it was confirmed that different factors contributed to suicidal ideation according to the SES group. In predicting suicidal ideation, the XGboost method predicted relatively better than the random forest method.

This study identified the risk factors of SI among adolescents and their association with SES. First, the basic analysis confirmed that low SES was strongly associated with SI [23,24]. This result is consistent with that of existing research. These results can be explained by the social causation hypothesis, which states that the income level of individuals and households affects people’s psychopathology [25]. According to this hypothesis, individuals with a low SES experience more adversity in their lives, and their stressful environment causes depression, anxiety, and post-traumatic stress disorder. The results of our study are consistent with this hypothesis, in that a decreasing SES level in this study was associated with increasing anxiety and behaviors such as drinking and smoking.

Regardless of SES level, people are equally likely to experience psychological problems, but individuals with a low SES may experience lower recovery rates because, unlike individuals with moderate or high SES levels, they lack access to treatment or resources to help them in difficult situations. Consequently, the prevalence of mental disorders was higher in the low SES group.

Previous studies have confirmed that children and adolescents with an upbringing in a low SES environment experience more emotional and behavioral issues such as anxiety, depression, physical symptoms, accidents, social withdrawal, aggression, and work and attention disorders [25]. Therefore, if the results of this study are interpreted according to age and SES level stratification, resolving these aforementioned emotional disorders in adolescence is challenging given they have a basis in childhood experiences.

Therefore, the low SES group might require support and preventive care through social and medical approaches much before adolescence. Such preventive approaches could be direct medical services; however, improving income through social access could be more effective. A long-term follow-up study including Native American Indian tribes confirmed that increasing their income not only alleviated poverty but also significantly minimized behavioral disorders among their children [26].

Other studies have shown that a change in psychological support resources during early adulthood affects the association between SES and distress symptoms [27]. That is, if psychological support resources are limited, the difference in symptoms between high and low SES levels is large; however, with increasing psychological support resources, the difference in symptoms according to SES decreases. These results also indicate differences in the possibility of a low SES individual experiencing psychological difficulties, depending on the extent of their access to psychological support resources.

In high SES group had relatively low positive predictive values compared to other groups. Previous studies also had low positive predictive values of 0–48% [20]. This is probably due to the low suicide rate. In this study, the prevalence of suicidal ideation was 8.63% in the high SES group, 10.81% in the middle group, and 24.31% in the low group. This is thought to be due to the lower prevalence of suicidal thoughts in the high SES group.

According to the machine learning based approach, in the medium and high SES groups, stress had the strongest association with SI, followed by sadness and anxiety. However, in the low SES group, anxiety had the strongest association with SI followed by anxiety. However, in the low SES group, relative sadness—that is, depression—did not contribute significantly to suicide risk.

Comparing SES groups revealed that the low SES group was about twice as high as the other groups. Contrarily, academic achievement also significantly influenced suicide risk in the high SES group but not in the low SES group.

Although further research is necessary to confirm these results, our study establishes that various factors in the high SES group and stress in the low SES group contributed to SI. Although we could not identify the causes of stress, we hypothesize that economic constraints were the primary reason in the lower SES groups and that the causes were more diverse among participants in the higher SES group. Access to psychological support resources varies depending on one’s SES level, and the SES level may affect one’s choice of support activity. Future studies should identify risk factors for stress and confirm the effect of the diversity of these factors on emotional states such as SI.

This study had limitations. First, the cross-sectional design and self-reporting based data did not allow evaluation of the long-term effect of SES. Second, the history or prevalence of mental illness could not be directly assessed. Therefore, future research should consider a comprehensive evaluation, including assessing prevalence, across cohorts. Third, we only considered SI in the past year as a binary variable rather than considering its severity. SI can be accidental, temporary, or passive, and which may significantly differ characteristically from active and continuous SI. Forth, the data used in this study were sampled to represent the country, but the weight was not adjusted during the analysis process.

Despite its limitations, this study identified risk factors for SI among adolescents by SES. A distinct strength of this study was the use of a large sample of the nationwide population, made possible by machine learning techniques. Additional research is needed to determine the effect of SES on the emotions of adolescents from various countries using prospectively collected data.

Although the variables used in this study have been identified as existing risk factors for suicide, new risk variables can be found if social networking service data or sensor data collected by smartphones is utilized [20]. In addition, for prediction using machine learning, analysis using real-time data can be attempted. Therefore, it will be utilized not only for the identification of variables using various data but also for efficient prediction.

## 5. Conclusions

Adolescents with suicidal thoughts experienced more sadness, more stress, less happiness, and more anxiety than other adolescents. Although SI was also observed in the high SES group, the low SES group showed the strongest association with emotional risk factors such as stress and anxiety. Therefore, implementing policies to improve adolescents’ income can be the foundation for improving their emotional health and ensuring their safety.

## Figures and Tables

**Table 1:** General characteristics of the subject by suicidal ideation.

	Total		Suicidal Ideation				*p* Value
			No		Yes		
Gender							<0.001
Male	28,353	51.6	26,099	53.3	2254	37.7	
Female	26,595	48.4	22,870	46.7	3725	62.3	
Sadness or hopelessness over 2 weeks							<0.001
No	41,108	74.8	39,468	80.6	1640	27.4	
Yes	13,840	25.2	9501	19.4	4339	72.6	
Perceived stress level in daily life							<0.001
Extremely	4603	8.4	2785	5.7	1818	30.4	
Stressful	14,059	25.6	11,423	23.3	2636	44.1	
Moderately	24,379	44.4	23,055	47.1	1324	22.1	
Minimally	9889	18.0	9734	19.9	155	2.6	
Not at all	2018	3.7	1972	4.0	46	0.8	
Feeling of happiness							<0.001
Very happy	15,111	27.5	14,666	29.9	445	7.4	
A little happy	20,064	36.5	18,785	38.4	1279	21.4	
Normal	14,960	27.2	12,880	26.3	2080	34.8	
A little unhappy	4070	7.4	2377	4.9	1693	28.3	
Very unhappy	743	1.4	261	0.5	482	8.1	
Violence victimization							<0.001
No	54,229	98.7	48,530	99.1	5699	95.3	
Yes	719	1.3	439	0.9	280	4.7	
GAD-7 score	3.91 ± 4.37		3.30 ± 3.78		8.84 ± 5.60		<0.001
Subjective health status							<0.001
Very good	15,150	27.6	14,244	29.1	906	15.2	
Good	23,294	42.4	21,151	43.2	2143	35.8	
Fair	12,342	22.5	10,543	21.5	1799	30.1	
Poor	3891	7.1	2876	5.9	1015	17.0	
Very poor	271	0.5	155	0.3	116	1.9	
Alcohol consumption (month)							<0.001
none	49,056	89.3	44,247	90.4	4809	80.4	
2 days	3495	6.4	2863	5.8	632	10.6	
3~4 days	1059	1.9	849	1.7	210	3.5	
6 days or more	1338	2.4	1010	2.1	328	5.5	
Smoking (month)							<0.001
Non-smoker	52,478	95.5	47,046	96.1	5432	90.9	
1~9 days	1168	2.1	898	1.8	270	4.5	
10 days or more	1302	2.4	1025	2.1	277	4.6	
Sexual experience							<0.001
No	52,461	95.5	47,050	96.1	5411	90.5	
Yes	2487	4.5	1919	3.9	568	9.5	
Drug abuse							<0.001
No	54,543	99.3	48,738	99.5	5805	97.1	
Yes	405	0.7	231	0.5	174	2.9	
Academic performance							<0.001
High	6736	12.3	6081	12.4	655	11.0	
Medium high	13,410	24.4	12,123	24.8	1287	21.5	
Medium	16,585	30.2	15,034	30.7	1551	25.9	
Medium low	12,684	23.1	11,150	22.8	1534	25.7	
Low	5533	10.1	4581	9.4	952	15.9	
Socioeconomic status							<0.001
High	6039	11.0	5518	11.3	521	8.7	
Medium	47,634	86.7	42,486	86.8	5148	86.1	
Low	1275	2.3	965	2.0	310	5.2	

*p* value by chi-square test and *t* test.

**Table 2:** Confusion matrix and prediction scores of XGBoost models by socioeconomic status.

Machine Learning Methods		Model	Sensitivity	Specificity	Accuracy	Positive Predictive Value	Negative Predictive Value	F1 Score	AUC
			(%)	(%)	(%)	(%)	(%)		
XGBoost model	Test data	High SES	57.9	81.6	79.4	24.3	95.0	0.343	0.773
		Middle SES	76.5	78.0	77.8	29.9	96.4	0.430	0.846
		Low SES	60.7	80.4	74.9	54.5	84.1	0.575	0.781
	Training data	High SES	69.8	80.4	79.4	26.1	96.4	0.380	0.835
		Middle SES	77.3	78.3	78.2	30.6	96.5	0.439	0.857
		Low SES	74.9	80.0	78.8	54.4	90.9	0.630	0.871
Random Forest	Test data	High SES	35.6	87.7	83.0	22.1	93.3	0.273	0.767
		Middle SES	52.0	84.9	81.4	29.3	93.6	0.375	0.794
		Low SES	56.3	80.8	74.6	49.5	84.6	0.526	0.762

**Table 3:** General characteristics of the subject by socioeconomic status.

	Total		Socioeconomic Status						*p* Value
			High		Medium		Low		
	N	(%)	N	(%)	N	(%)	N	(%)	
Suicidal ideation	5979	10.9	521	8.6	5148	10.8	310	24.3	
Gender									<0.001
Male	28,353	51.6	3536	58.6	24,095	50.6	722	56.6	
Female	26,595	48.4	2503	41.4	23,539	49.4	553	43.4	
Sadness									<0.001
No	41,108	74.8	4689	77.6	35,701	74.9	718	56.3	
Yes	13,840	25.2	1350	22.4	11,933	25.1	557	43.7	
Perceived stress									<0.001
Extremely	4603	8.4	515	8.3	3804	8.0	284	22.3	
Stressful	14,059	25.6	1191	19.7	12,470	26.2	398	31.2	
Moderately	24,379	44.4	2429	40.2	21,512	45.2	438	34.4	
Minimally	9889	18.0	1364	22.6	8404	17.6	121	9.5	
Not at all	2018	3.7	540	8.9	1444	3.0	34	2.7	
Feeling of happiness									<0.001
Very happy	15,111	27.5	2816	46.6	12,081	25.4	214	16.8	
A little happy	20,064	36.5	1785	29.6	17,975	37.7	304	23.8	
Normal	14,960	27.2	1101	18.2	13,426	28.2	433	34.0	
A little unhappy	4070	7.4	263	4.4	3577	7.5	230	18.0	
Very unhappy	743	1.4	74	1.2	575	1.2	94	7.4	
Violent victimization									<0.001
No	54,229	98.7	5903	97.7	47,103	98.9	1223	95.9	
Yes	719	1.3	136	2.3	531	1.1	52	4.1	
GAD-7 score	3.91 ± 4.37		3.12 ± 4.30		3.95 ± 4.31		6.02 ± 5.86		<0.001
Subjective health status									<0.001
Very good	15,150	27.6	2711	44.9	12,130	25.5	309	24.2	
Good	23,294	42.4	2205	36.5	20,705	43.5	384	30.1	
Fair	12,342	22.5	848	14.0	11,125	23.4	369	28.9	
Poor	3891	7.1	234	3.9	3478	7.3	179	14.0	
Very poor	271	0.5	41	0.7	196	0.4	34	2.7	
Alcohol consumption (month)									<0.001
No drinker	49,056	89.3	5431	89.9	42,597	89.4	1028	80.6	
2 days	3495	6.4	307	5.1	3076	6.5	112	8.8	
3~4 days	1059	1.9	100	1,7	913	1.9	46	3.6	
6 days or more	1338	2.4	201	3.3	1048	2.2	89	7.0	
Smoking (month)									<0.001
Non-smoker	52,478	95.5	5737	95.0	45,605	95.7	1135	89.0	
1~9 days	1168	2.1	123	2.0	992	2.1	53	4.2	
10 days or more	1302	2.4	179	3.0	1036	2.2	87	6.8	
Sexual experience									<0.001
No	52,461	95.5	5668	93.9	45,659	95.9	1134	88.9	
Yes	2487	4.5	371	6.1	1975	4.1	141	11.1	
Drug abuse									<0.001
No	54,543	99.3	5978	99.0	47,320	99.3	1245	97.6	
Yes	405	0.7	61	1.0	314	0.7	30	2.4	
Academic performance									<0.001
High	6736	12.3	1907	31.6	4739	9.9	90	7.1	
Medium high	13,410	24.4	1608	26.6	11,664	24.5	138	10.8	
Medium	16,585	30.2	1247	20.6	15,129	31.8	209	16.4	
Medium low	12,684	23.1	816	13.5	11,502	24.1	366	28.7	
Low	5533	10.1	461	7.6	4600	9.7	472	37.0	

The *p* value by chi-square test and ANOVA test.

## Data Availability

Data may be requested form the corresponding author.
